# Machine-Learning Classification of Soil Bulk Density in Salt Marsh Environments

**DOI:** 10.3390/s21134408

**Published:** 2021-06-27

**Authors:** Iman Salehi Hikouei, S. Sonny Kim, Deepak R. Mishra

**Affiliations:** 1 Appalachian Laboratory, University of Maryland Center for Environmental Science, Frostburg, MD 21532, USA; iman.salehihikouei@umces.edu; 2 College of Engineering, University of Georgia, Athens, GA 30602, USA; 3 Department of Geography, University of Georgia, Athens, GA 30602, USA; dmishra@uga.edu

**Keywords:** soil characterization, random forest, XGBoost, machine learning, coastal wetlands, Landsat-7 (ETM+)

## Abstract

Remotely sensed data from both in situ and satellite platforms in visible, near-infrared, and shortwave infrared (VNIR–SWIR, 400–2500 nm) regions have been widely used to characterize and model soil properties in a direct, cost-effective, and rapid manner at different scales. In this study, we assess the performance of machine-learning algorithms including random forest (RF), extreme gradient boosting machines (XGBoost), and support vector machines (SVM) to model salt marsh soil bulk density using multispectral remote-sensing data from the Landsat-7 Enhanced Thematic Mapper Plus (ETM+) platform. To our knowledge, use of remote-sensing data for estimating salt marsh soil bulk density at the vegetation rooting zone has not been investigated before. Our study reveals that blue (band 1; 450–520 nm) and NIR (band 4; 770–900 nm) bands of Landsat-7 ETM+ ranked as the most important spectral features for bulk density prediction by XGBoost and RF, respectively. According to XGBoost, band 1 and band 4 had relative importance of around 41% and 39%, respectively. We tested two soil bulk density classes in order to differentiate salt marshes in terms of their capability to support vegetation that grows in either low (0.032 to 0.752 g/cm^3^) or high (0.752 g/cm^3^ to 1.893 g/cm^3^) bulk density areas. XGBoost produced a higher classification accuracy (88%) compared to RF (87%) and SVM (86%), although discrepancies in accuracy between these models were small (<2%). XGBoost correctly classified 178 out of 186 soil samples labeled as low bulk density and 37 out of 62 soil samples labeled as high bulk density. We conclude that remote-sensing-based machine-learning models can be a valuable tool for ecologists and engineers to map the soil bulk density in wetlands to select suitable sites for effective restoration and successful re-establishment practices.

## 1. Introduction

Salt marshes are ecologically sensitive ecosystems that connect the terrestrial and marine environments and serve as critical habitats for flora and fauna [1,2,3,4,5,6,7]. Salt marshes are negatively impacted by anthropogenic exploitation in the form of coastal development and natural resource extraction [4,6,8,9]. Disturbances cause irreversible alterations in the condition of salt marsh communities over time [10,11,12,13,14,15]. Sea-level rise, drought, and physical alteration in soils, as well as changes in hydrological patterns, exert major pressures on salt marsh ecosystems [16,17,18,19,20]. Disturbances in salt marshes negatively impact soil structure, which is one of the main components of coastal wetlands and responsible for the high primary production of coastal marshes [21,22,23,24]. Bulk density increases due to disturbances accelerating organic matter degradation, compaction, and erosion [23,25], and as a result, soil volume decreases [26]. Compaction yields an increase in the fraction of soil pores filled with water at a constant moisture content level as the average pore size decreases [27]. An increase in soil bulk density changes the soil aeration properties [28], alters soil biological processes due to a decrease in soil temperature [29], expedites the soil denitrification process [30], causes loss in the mycorrhizal fungi community [31], and restricts the vegetation root growth [32]. Bulk density is typically measured for characterizing soil structure and utilized for measuring total porosity [33]. Bulk density reflects soil’s structural stability to support vegetation growth against the destructive impacts of tidal flooding; however, bulk density greater than 1.6 g/cm^3^ is not suitable for root and plant growth in salt marshes [34]. Studies have shown that an increase in soil bulk density from 1.1 to 1.4 g/cm^3^ yielded a 42% reduction in oxygen diffusion rate through waterlogged salt marsh soil, while the induced changes in soil bulk density from 1.1 to 1.7 g/cm^3^ resulted in a 75% reduction in the rate of oxygen diffusion [35]. Within a given type of soil texture, variations in bulk density are directly related to the degree of compactness [36], aggregation [37], and organic matter content [38].

In wetlands, bulk density is a fundamental parameter influencing hydraulic conductivity [37,39], and this parameter is used for calculating the total storage of a given nutrient per unit area [2,40]. Nutrient stock (carbon or nitrogen) in a wetland environment could be estimated by soil bulk density, and soils with low bulk density have a greater capacity for holding nitrogen and carbon than soils with high bulk density [26]. For carrying out long-term monitoring of the health and biophysical status of salt marshes and detecting alterations in their soil structures, it is helpful to access and utilize up-to-date sources of salt marsh spatial extent data, which are typically remotely sensed at a broad scale.

Point-collection methods for determining soil properties at a large salt marsh site do not yield results that accurately reflect the soil structure and function of the entire area because salt marshes have high spatial variabilities in soil conditions, vegetation communities, hydrological patterns [41,42]. Furthermore, traditional soil analyses are based on procedures requiring in-situ sampling and subsequent laboratory processing [43]. Field sampling requires a considerable amount of time and effort and may not be cost-effective for the long-term monitoring practices of a large study area [43]. Remote-sensing techniques are increasingly being used as rapid, cost-effective, and nondestructive approaches to modeling soil properties at a large scale [43,44,45,46,47,48].

Visible near-infrared (VNIR) and shortwave infrared (SWIR) reflectance from soil surfaces include information that can be used for determining the qualitative and quantitative properties of soil structure [49]. VNIR and SWIR spectroscopy for determining soil properties is founded upon the vibrations of chemical bonds in soil molecules [50]. In the visible region (400–700 nm), electronic transitions produce wide absorption bands corresponding to chromophores that influence soil color; whereas in the NIR–SWIR (700–2500 nm), bending of the N-H, O-H, and C-H bonds lead to weak overtones and vibrations [51,52]. Laboratory NIR measurements illustrated that OH groups have strong absorption features at the regions of 1400–1900 nm, mainly because of soil water content, hydroxyls, and clay content [53]. Using these types of remotely sensed data of soils, or vegetation plus soils, and techniques such as time-series analysis of vegetation indices, studies have identified soil types and changes in soil structure [41,45]. Soil properties in tidal wetlands have been linked to vegetation density, diversity, and health [54], and using similar remote-sensing techniques, soil properties have also been characterized based on the composite spectral reflectance from the salt marsh surface, which includes the reflectance from moist background soil and the reflectance from vegetation canopy [44]. Salt marsh soil properties such as salinity, organic matter, and moisture content have been investigated by using remote sensing (hyperspectral imagery) and machine-learning algorithms [44,55]. Although [29] reported that hyperspectral data are more accurate than multispectral data in characterizing salt marsh soil properties due to their fine spectral resolution, multispectral data are cost-effective and allow for long-term time-series analysis of salt marsh soil properties. According to their study, because of the high temporal variability of soil salinity and water content, low spatiotemporal variation of soil organic matter, and time lag of vegetation response to changes occurring in soil properties, direct applications of soil characterization models derived from time-series analysis of hyperspectral data were problematic. Therefore, it is necessary to conduct more research for recalibrating time intervals for soil characterization models that depend upon the temporal variability of soil properties and vegetation structures at a salt marsh site.

This paper investigates the utility of Landsat-7 (ETM+) data to estimate salt marsh soil bulk density using machine-learning algorithms. To date, use of remote-sensing data for estimating salt marsh soil bulk density at the vegetation rooting zone has not been investigated or published. Machine-learning algorithms as a nonparametric method outperform parametric statistical models in estimating soil attributes at rooting depth because parametric models are only limited to the few first centimeters of the topsoil [52]. Furthermore, machine-learning models minimize the interference from the soil moisture content and vegetation canopy of a marsh surface, leading to more accurate prediction of soil properties than parametric models [52]. The main objective of this study is to evaluate the capability of freely accessible multispectral Landsat-7 data for estimating salt marsh soil bulk density by comparing the performance of random forest (RF), super vector machine (SVM), and extreme gradient boosting (XGBoost) models and rank the most important spectral bands for bulk density estimation. In this study, the spectral bands of Landsat-7 (ETM+) are prioritized for predicting soil bulk density at a salt marsh site. Although some previous studies have reported that VNIR and SWIR are highly recommended for estimating soil moisture content and organic matter content [50,56,57], the efficiency of those spectral bands in predicting soil bulk density in a salt marsh environment has not been explored. Furthermore, this study also investigates the importance of the vegetation indices such as NDVI, RVI, and DVI in classifying salt marsh soil bulk density. Remote-sensing-based machine-learning models for soil bulk density mapping should be assessed and validated prior to utilization because a reliable tool can be invaluable for selecting suitable sites for effective restoration and successful re-establishment practices. Therefore, the accuracy of machine-learning algorithms in governing the importance of the Landsat-7 (ETM+) spectral bands as well as the vegetation indices for predicting soil bulk density is comprehensively evaluated in this paper.

## 2. Materials and Methods

### 2.1. Method Summary and Data Used

Salt marshes along Georgia’s Atlantic coast in the US were selected for this study. Georgia has the second-largest geographic area of salt marshes in the US [58]. Georgia’s coastal marshes encompass approximately 378,000 acres in a four-to-six-mile band behind the barrier islands. These marshes have been identified as one of the most extensive and productive ecosystems in the United States [58]. Nearly 286,000 acres of these marshes are covered by a salt-tolerant species of marsh grass, known as *S. alterniflora* or smooth cordgrass [58]. The remaining 107,000 acres support other types of salt, brackish, and freshwater marshes.

**Figure 1:**
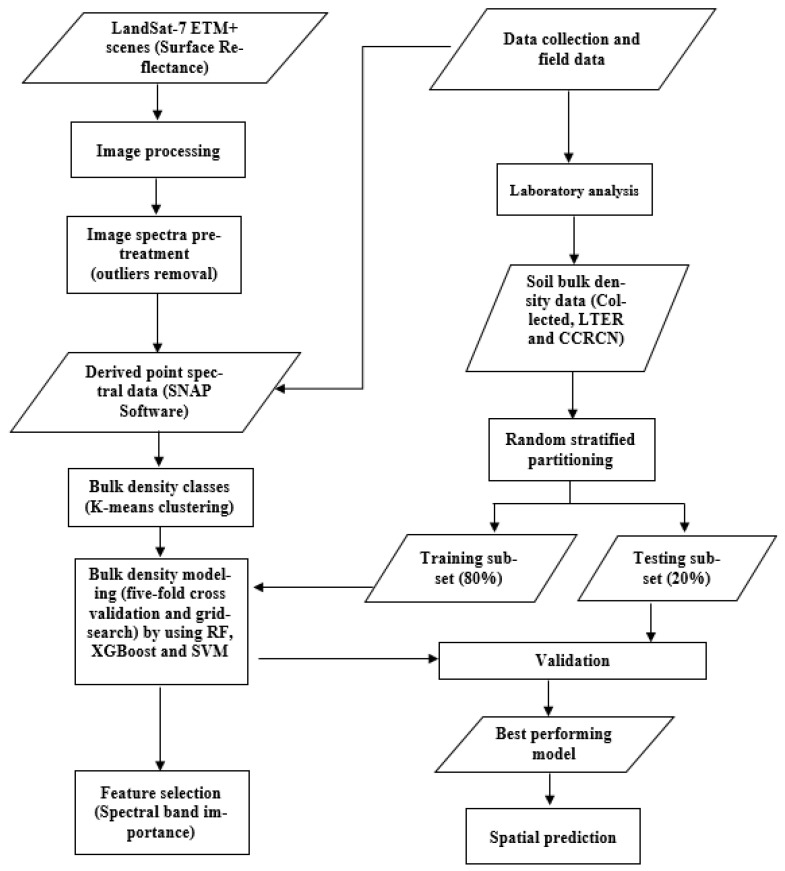
Flowchart illustrating the steps for soil bulk density prediction using Landsat-7 ETM+ data.

Figure 1 shows the methodology steps for estimating salt marsh soil bulk density by using remote-sensing data and machine-learning algorithms. Data used in the methodology include Landsat-7 Enhanced Thematic Mapper Plus (ETM+) multispectral data, and soil data collected by field sampling and laboratory analysis. Landsat-7 (ETM+) surface reflectance data with a spatial resolution of 30 m are available from the US Geological Survey (USGS) Earth Resources Observation and Science Center [59], and approximate scene size of 170 km north–south by 183 km east–west, covering the area of interest. Landsat-7 (ETM+) images corresponding to the sampling salt marshes were obtained and processed over the study period (i.e., sampling date from 2000 to 2018 inclusive, Table 1). Band 6, the thermal band (10.40–12.50 µm), was not used in this study. Clouds were nearly absent in the acquired Landsat-7 (ETM+) data, and the reflectance values were extracted from the field sampling locations using ESA’s SNAP (the European Space Agency’s Sentinel Application Platform, version 7.0) software.

The bulk density datasets were collected and archived by the Coastal Carbon Research Coordination Network (CCRCN), hosted at the Smithsonian Environmental Research Center. CCRCN is an initiative to expedite the pace of scientific discovery in coastal wetlands by providing the community with access to data, open-source analysis tools, and data synthesis opportunities. The sampling marshes were located and distributed along Georgia’s coastline, from Wilmington Island to Cumberland Island. These datasets were downloaded from the Coastal Carbon Atlas, a map interface that accesses the CCRCN’s data library [60]. Each data source was credited to the original data contributors [61,62,63,64,65,66]. The rest of the bulk density data was credited to the original data contributor [67,68] and obtained from the Georgia Coastal Ecosystems Long-Term Ecological Research (GCE LTER) program [69].The GCE LTER project aims to understand the patterns and processes that shape change in estuarine and marsh environments. A total of 346 salt marsh plots (1 × 1 m) were surveyed along 24 transects, and root-zone soil samples were cored over marsh regions for laboratory analysis. The location of each plot was recorded using a GPS unit.

The spectral indices such as NDVI (Normalized Difference Vegetation Index), RVI (Ratio Vegetation Index), and DVI (Difference Vegetation Index) were derived from the Landsat-7 ETM+ images recorded during our study period and calculated as follows:

NDVI = (NIR − RED)/(NIR + RED)(1)

RVI = NIR/RED(2)

DVI = NIR−RED(3)

where NIR and RED are near-infrared (Band 4) and red (Band 3), respectively. These indices, as well as Landsat-7 ETM+ spectral bands, were employed as independent variables for estimating soil bulk density. The independent variables used in this study include band 1 through 5, band 7, NDVI, RVI, and DVI.

In addition to the above data sources, sampling occurred in eight tidal marshes along the southeast coast of the US in Georgia in 2018. Three different representative sampling areas were chosen based on vegetation coverage typologies and hydroperiods. For each distinct vegetation community, species richness and number of individuals were estimated utilizing the cover scale of Braun-Blanquet [70]. The predominant vegetative species were characterized in accordance with the vegetation survey conducted at all sampling sites. Transects began several meters inside the marsh so that all samples were representative of the marsh itself, not the upland border. Furthermore, the soil coring method was applied for measuring soil bulk density at the root zone [33]. A soil sampler was utilized to collect an undisturbed soil sample from the root zone to determine the bulk density at the laboratory. The soil coring procedure is the most common method utilized to measure soil bulk density [71]. The core sampling tests were carried out based on the procedure described in [72]. In this procedure, a solid ring cylinder was gently pressed into the soil to take a core sample [73]. We excavated around the ring without disturbing or loosening the soil it contained and carefully removed it with the soil intact. We removed any excess soil from the outside the ring and cut any plants or roots off at the soil surface with scissors. Soil samples were collected in the rooting zone at the sites and kept intact in sealed waterproof containers to avoid moisture loss. All samples were transported to the laboratory within four hours and stored at 4 °C for the measurement of soil bulk density. Samples were dried at 105 °C for 2 days, and then the mass of dry soil samples, as well as their bulk density, were determined. The total volume of the soil was calculated as the internal volume of the cylinder. Soil bulk density was calculated as follows:

Bulk Density = Dry Soil Weight/Soil Volume(4)

where bulk density, dry soil weight, and soil volume are in (g/cm^3^), (g), and (cm^3^), respectively. The general data descriptions, including data source, sampling date, sample number, minimum, maximum, average, and standard deviation for each dataset were determined (Table 1).

In this study, although we performed a quantitative machine-learning analysis to predict soil bulk density using Landsat7 ETM+ spectral bands as independent variables, the resulted R square was lower than 0.05. Due to a very low R square, the results from the regression analysis are not reported in this paper. Thus, we carried out a classification analysis with machine-learning classification algorithms using Python (version 3.7) to obtain acceptable results for estimating salt marsh soil bulk density.

### 2.2. Machine-Learning Algorithms

The K-means algorithm was employed for the bulk density data for determining cluster numbers, centers, and ranges. The K-means clustering method classifies a dataset into different clusters, including points with similar characteristics. In this method, each observation in the dataset is initially assigned to one of *k* clusters at random [74]. The centroid location is determined for each cluster, and then each point is re-assigned to a cluster with the nearest centroid [74]. This iteration process stops when there is no change in cluster membership with additional iterations of the algorithm [75].

After K-means clustering, three machine-learning algorithms, support vector machine (SVM), random forest (RF), and extreme gradient boosting (XGBoost) were implemented to determine the most accurate model for soil bulk density prediction in salt marsh environments using multispectral Landsat-7 surface reflectance data. Over the last two decades, RF, SVM, and XGBoost classifiers have received increasing attention because of their accurate classification results and considerable faster processing speed [76,77,78,79,80]. RF and XGBoost algorithms yield reliable classifications using predictions derived from an ensemble of decision trees [81,82,83]. Furthermore, these classifiers can be successfully used to select and rank those variables with the greatest ability to discriminate between the target classes [82,84,85]. This is an important asset given that the high dimensionality of remotely sensed data makes the selection of the most relevant variables a time-consuming, error-prone, and subjective task [82,84,86,87]. SVM is particularly appealing in remote sensing due to its ability to successfully handle the high dimensionality of remotely sensed data [78,88], often producing higher classification accuracy than the traditional methods [89,90,91]. The underlying principle that benefits SVM is the learning process, which follows what is known as structural risk minimization [92]. Under this scheme, SVM minimizes classification error on unseen data without prior assumptions made on the probability distribution of the data [92,93]. A number of studies have systematically investigated the utilization of these classifiers for remote-sensing data classification [58,59,60,61,62,63,64,65,66,67,68,69,70,71,72,73]. There has, however, been no publication to date dedicated to summarizing the application of these efficient machine-learning algorithms in classifying salt marsh soil bulk density and prioritizing spectral features based on their importance and contribution to soil bulk density prediction.

Overall, SVM is a binary classifier that transforms *n*-class problems into the sequence of binary classification tasks [94]. The basic variant of SVM produces a separating hyper-plane in the original space of *n* coordinates between the points of two distinct classes [95]. In SVM, the hyper-plane is built from the training set, determines a maximum margin of separation between the classes, and generates a classification hyper-plane in the middle of the maximum margin [95].

RF utilizes ensemble approaches based on calculating the average of a large number of separate decision-tree models built by finding the best predictor for splitting the results with consideration of the model error [96]. Overall, ensemble learning is defined as a method that makes predictions based upon several different models [97]. By amalgamating individual models (trees), the developed ensemble model minimizes the overfitting problem [97]. These RF trees are developed by bootstrapped training dataset, and only a small number of variables is chosen at one split, and as such, these generated trees do not have collinearity issues with each other [98].

The gradient boosting technique is used for developing boosted decision-tree models. In this method, the gradient boosting technique is used to fit the simple base learner functions of decision trees to the pseudo-residuals, which are the gradient of the minimized loss function through sequent iterations [99]. The boosted regression tree model exhibits more acceptable performance than other machine-learning techniques [100] and is specifically suitable for less-than-clean data [101]. Tree-based models such as RF and the boosted regression tree classify features based on their relative importance, as in the following equation:

(5) $${\hat{J{\;}_{i}}}^{2}=\sum_{Splits\;on\;X_{i}}I_{t}^{2}$$

The approximate relative influence (${\hat{J_{i}}}^{2}$) of a predictor variable x*_i_* was calculated by the equation above, where $I_{t}^{2}$ is the empirical improvement by splitting on predictor 𝑥_i_ at that point.

A feature relative importance assessment was carried out to evaluate the relative importance of each feature (i.e., spectral bands) in predicting the target variable, soil bulk density. The relative importance of a feature, utilized as a decision node in a decision tree, was calculated to determine the predictability of independent variables [102]. Features at the top of the tree contribute to the final prediction decision of a greater fraction of the input samples. The fraction of the samples that a feature contributes to is used to estimate its relative importance. The machine-learning feature selection technique, based on the feature’s relative importance, has been used in soil science to determine the most important parameters for soil organic matter [103,104]. In this study, XGBoost and RF as ensemble machine-learning algorithms were employed to investigate the most important spectral bands for bulk density prediction.

### 2.3. Model Training and Assessment

We randomly selected 75% (744) of the soil bulk density data as a training dataset to predict salt marsh soil bulk density by using Landsat-7 spectral bands as the only inputs. The *k*-fold cross-validation method (*k* = 5) was used as a resampling procedure to improve the effectiveness of machine-learning models. For the validation purpose, the fitted model from the training dataset was employed for predicting the testing subset with consideration of the calculated error rate. By using a five-fold cross-validation technique, the dataset considered for the training part was randomly segmented into five equal subsets, and the fitting process was repeated five times by using a different subset as the validation subset. The optimal parameters on the given dataset need to be determined in order to make the best classification model. For this purpose, we used an exhaustive grid search for determining the optimal kernel of poly, kernel coefficient (γ), and regularization parameter (C) for the SVM space as suggested in [105], the minimum number of samples required for each leaf, the minimum number of samples required to split each node, the maximum number of levels in each decision tree, the number of trees in the forest for RF as suggested in [23,83], and the number of trees in the ensemble, a maximum tree depth, and a learning rate for XGBoost as suggested in [83].

The model assessment was carried out by using a testing subset (*n* = 248) which was not considered for the model training. In other words, the remaining 25% of the dataset was used for validation. The most common method to assess the classification accuracy of remotely sensed data is the confusion matrix. A confusion matrix is a square array of numbers set out in rows and columns, which expresses the relationship between the actual soil bulk density class in the reference and the predicted soil bulk density class. A confusion matrix was constructed for evaluating machine-learning algorithms’ efficiency and accuracy in classifying salt marsh soil bulk density. Classification accuracy is defined as a number of data points in the testing dataset correctly classified as high- and low-bulk-density classes divided by testing dataset size. Precision for the high-bulk-density class is the number of true positives (i.e., the number of items correctly labeled as high-bulk-density class) divided by the total number of elements labeled as belonging to this class (i.e., the sum of true positives and false positives, which are items incorrectly labeled as belonging to the class). On the other hand, precision for the low-bulk-density class is the number of the items correctly classified as low-bulk-density class divided by the total number of elements that are placed in this class. Recall for the high-bulk-density class is defined as the number of items correctly labeled divided by the total number of elements that actually belong to this class. However, recall for the low-bulk-density class is defined as the number of items correctly classified divided by the total number of elements that actually belong to this class.

## 3. Results

### 3.1. K-Means Algorithm for Data Labeling Based on Bulk Density and Salt Marsh Species

**Figure 2:**
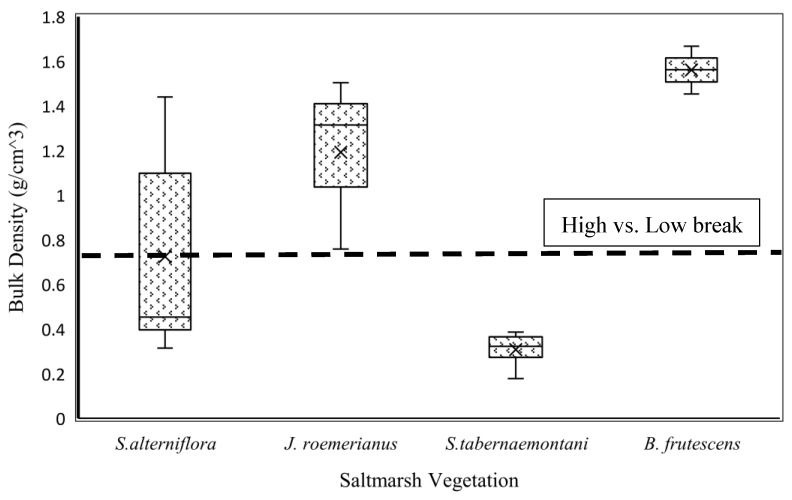
Bulk density and salt marsh vegetation types.

The K-means clustering algorithm was used to cluster bulk density into two classes: low and high bulk densities, ranging from 0.032 g/cm^3^ to 0.752 g/cm^3^ and 0.752 g/cm^3^ to 1.893 g/cm^3^, respectively. The cluster center for the low-bulk-density class was 0.400 g/cm^3^, which tends to be suitable for supporting salt marsh vegetation with a soft root structure such as *Schoenoplectus tabernaemontani* [26]. The center for the high-bulk-density class was 1.108 g/cm^3^, which is suitable for salt marsh vegetation such as *Juncus roemerianus* and *Borrichia frutescens* [26]. Therefore, 0.752 g/cm^3^ is considered a crucial threshold for detecting and distinguishing a salt marsh vegetation type with different root structures. This matter is considerably important for conducting an efficient restoration practice and a successful vegetation re-establishment. Vegetation surveys and bulk density experiments were conducted along the Georgia coast at 24 salt marsh sites in June 2018 to determine the importance of bulk density in salt marsh vegetation diversity. Figure 2 shows that bulk density plays a key role in vegetation diversity. For example, *S. tabernaemontani* grows in soils with low bulk density, while *B. frutescens* and *J. roemerianus* are able to develop and establish in soils with high bulk density. According to [26], the salinity range for *B. frutescens*, *J. roemerianus, S. alterniflora,* and *S. tabernaemontani* are (5.44, 32.57), (12.28, 22.88), (23.61, 32.14) and (2.83, 4.73), respectively.

### 3.2. Band Selection for Modeling Soil Bulk Density

**Figure 3:**
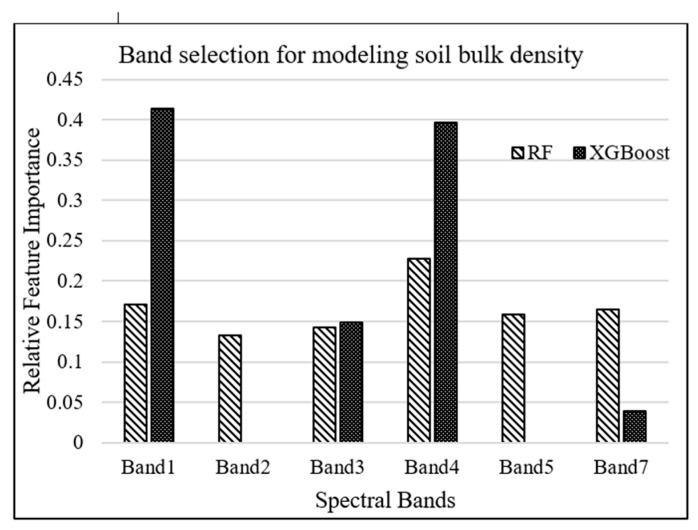
Relative importance of Landsat-7 bands for modeling bulk density.

RF and XGBoost algorithms were used to investigate the most important Landsat-7 (ETM+) spectral bands for modeling salt marsh soil bulk density. The tuning hyperparameters for RF model, including the minimum number of samples required for each leaf, the minimum number of samples required to split each node, the maximum number of levels in each decision tree, and the number of trees in the forest, are chosen to be 4, 6, 3, and 500, respectively, based on an exhaustive grid search used to find the optimal hyperparameters of a model in order to achieve the most accurate predictions. On the other hand, the XGBoost model was tuned with the hyperparameters of 100 trees in the ensemble, a maximum tree depth of 3, and a learning rate of 0.5. As shown in Figure 3, band 1 (blue) and band 4 (near-infrared) were estimated as the most important attributes for modeling bulk density by XGBoost and RF algorithms, respectively.

**Figure 4:**
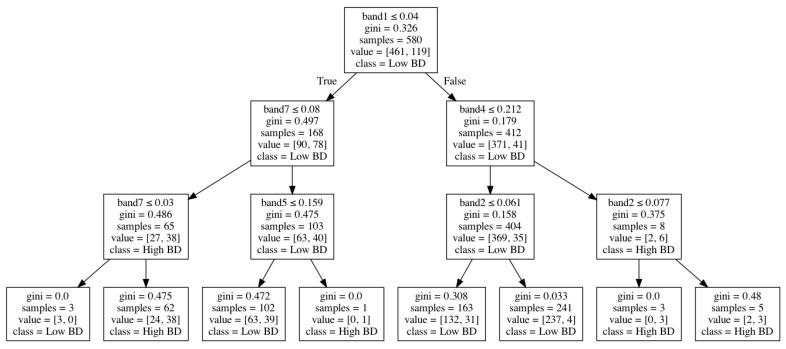
An example of the decision tree for bulk density classification.

Figure 4 shows how bulk density was modeled through a decision-tree algorithm based on Landsat-7 spectral bands. In this tree structure, an internal node represents a “test” on an attribute (e.g., Landsat-7 spectral bands), a branch represents the output of the test, a leaf node represents a class label (low or high bulk density), and the paths from the root to leaf represent classification rules.

**Figure 5:**
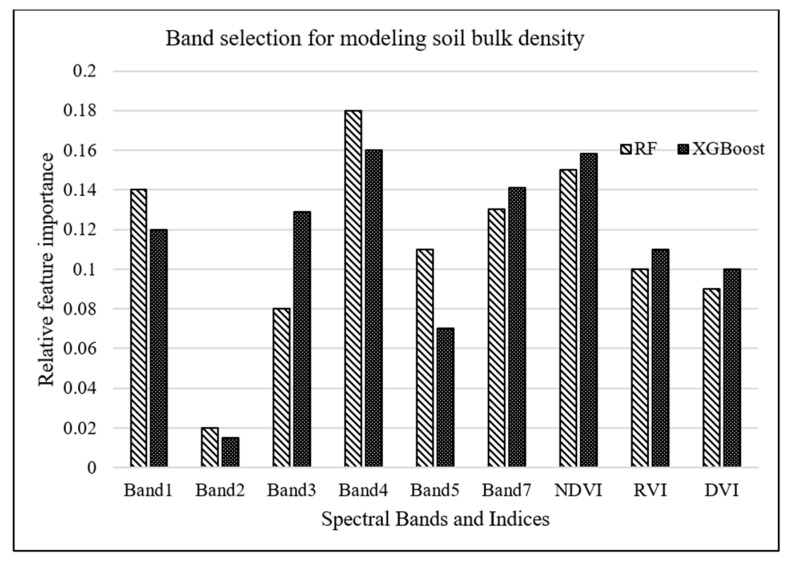
Relative importance of LandSat-7 bands as well as vegetation indices for modeling bulk density.

A complementary analysis was performed to investigate the importance of spectral vegetation indices in predicting soil bulk density through employing RF and XGBoost algorithms. Results of this analysis, demonstrated in Figure 5, concluded that NDVI was the second most important parameter for bulk density estimation. However, adding these indices resulted in no improvement in the prediction accuracy of the machine-learning models because vegetation indices, which are a combination of different spectral bands, do not introduce a new dimension, i.e., additional to the spectral bands, into machine-learning algorithms.

### 3.3. Soil Bulk Density Prediction

XGBoost and RF as ensemble tree models were employed to assign the salt marsh soils into two main classes as low and high soil bulk densities. This classification was carried out by only using Landsat-7 (ETM+) spectral band values as independent variables. Table 2 shows that XGBoost had the highest accuracy of 0.88 and the lowest MSE of 1.26 among the study algorithms. According to RF, low- and high-bulk-density classes had the precision of 0.96 and 0.62, respectively, meaning that once the RF algorithm assigns a low-bulk-density class to a salt marsh site, it is correct 96% of the time. On the other hand, the XGBoost model had precisions of 0.88 and 0.86, corresponding to low and high bulk densities, respectively. The RF model had a recall of 0.88 and 0.83, corresponding to low and high bulk densities, respectively. In other words, this algorithm correctly identified 88% of all low-bulk-density and 83% of all high-bulk-density salt marshes.

Table 3 demonstrates the classification outputs by the machine-learning algorithms on the test dataset (*n* = 248). This table shows that machine-learning models had a better performance in identifying the sampling sites with low bulk density than high bulk density. For example, XGBoost correctly identified 178 out of 186 of the low-bulk-density sites, while this algorithm accurately classified 39 out of 63 of the high-bulk-density marshes.

**Figure 6:**
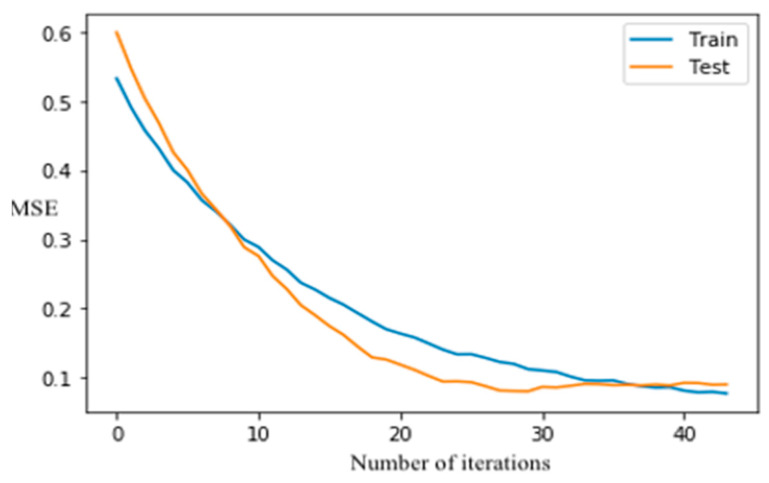
XGBoost classification error vs. the number of iterations.

The XGboost model did not overfit because the classification error in testing dataset reduces as the number of iterations increases, and the curves (Figure 6) are converged after 40-time runs once the learning rate is 0.5. The SVM algorithm was also employed on the study dataset. As shown in Table 2, the tuned SVM model had an accuracy of 0.86 on the testing dataset. Although XGboost had a slightly better performance than RF and SVM (Table 2), the difference in accuracy between each pair of these machine-learning methods was negligible. The training speed or computational cost of a machine-learning algorithm may be of concern, especially if a large number of training samples are used in developing a classification model. For operational applications of machine learning, which may involve huge data sets, prediction speed must be considered. The substantial drawback of the applicability of SVM is kernel selection. Although many options are available, some kernel functions may not provide optimal SVM configuration for remote-sensing applications [106]. The speed of SVM analysis was controlled by regularization parameter and kernel parameters, whereas the speed of XGboost was affected by selecting optimum tuning hyperparameters such as learning rate and the number of trees. A set of user-defined parameters were required to design the SVM model, and the hypermeters, including the kernel of poly, kernel coefficient (γ) of 40, and regularization parameter (C) of 1 were set to tune the model. The design of the SVM model involved selecting optimal kernels γ and C, which requires a lot of experimentation and processing time compared to RF and XGboost.

## 4. Discussion

We demonstrate that the open-sourced multispectral data, such as from the Landsat-7 ETM+, which was the only sensor operational during the whole study period, can be suitable for soil bulk density digital mapping. With more coincident training data from field sites, Landsat-5 and 8 datasets can also be trained using a similar machine-learning framework to create a significant long-term time-series bulk-density product for wetland ecosystems, a novel application of satellite data that is currently lacking.

### 4.1. Spectral Features for Salt Marsh Soil Bulk Density Prediction

The spectral resolution of the sensors significantly influences the quality of soil attributes prediction [52]. It is, therefore, necessary to utilize remote-sensing data with the appropriate spectral resolution taken across VNIR and SWIR spectrum for accurate soil bulk density predictions [52,107]. Our study also investigated the importance of bands in machine-learning prediction models. Band selection analysis underlined the general importance of a spectral band in soil bulk density prediction. According to our results, band 1 (450–520 nm) and band 4 (770–900 nm) of Landsat-7 ETM+ were selected as the most important parameters for modeling bulk density by XGBoost and RF algorithms, respectively. In bare soils, surface reflectance mainly carries soil features, while in salt marsh soils, surface reflectance is a composite signal and includes features from salt marsh surface elements such as vegetation canopy, water, and soil. According to [26], soil organic matter content is the most important parameter influencing soil bulk density. For example, if soil organic matter content decreases in a soil substrate, soil bulk density increases. Thus, spectral bands used for detecting soil organic matter and predicting its content can be helpful for characterizing soil bulk density. Soil organic matter decreases the spectral reflectance in VNIR region, especially if the soil organic matter content is greater than 2% [108,109]. That is because humic acid, the darkest pigment of soil organic matter, reduces the spectral reflectance in VNIR and SWIR [30]. Salt marsh soils generally contain organic matter higher than 2%, which can substantially affect the reflectance in VNIR region from the salt marsh surface. In the visible range (400–700 nm), the blue band is attributed to the electron transition of iron oxides [110]. This electron transition generates wide absorption bands related to chromophores that influence soil color, while in near-infrared (band 4), weak overtones and combinations of these vibrations occur due to stretching and bending of the C-H bonds [50], which mainly are found in organic compounds. Therefore, spectral reflectance in blue and near-infrared regions are considered as two essential spectral features for determining soil organic matter content, and as such, soil bulk density.

According to RF and XGBoost, Figure 5 demonstrates that NDVI is the second most important feature explaining bulk density variability, meaning that soil bulk density is highly influenced by vegetation variation and structure. Soil bulk density is a function of organic matter content [26]. Organic matter content and NDVI are highly dependent on the natural vegetation cover structure and the plant residue left after plant harvesting [111]. Therefore, organic matter content vividly explains the link between bulk density and NDVI. This study exhibits that NDVI has a greater importance to soil bulk density compared to other remotely sensed vegetation indices like RVI and DVI. Although the EVI and DVI perform better than the NDVI in many applications, our results indicated that NDVI is more important for explaining soil bulk density variability compared to RVI and DVI.

### 4.2. Machine-Learning Assessment for Soil Bulk Density Classification Using Remotely Sensed Data

The identification of surface soil features and land resources is important for precise management at different scales. Although spectral signatures related to some soil properties such as moisture content and salinity are influenced by spatial and temporal variability of surface processes, bulk density is less dependent on surface processing than soil moisture content or salinity, and it changes slowly over time. Since soil bulk density is characterized by slow temporal dynamics, salt marsh maps of soil bulk density are recommended to be generated every few years. However, the main limiting factor in soil characterization through remote-sensing spectral bands is using an appropriate procedure for finding the optimal correlation between soil reflectance data and soil bulk density. Using machine-learning algorithms such as RF, XGboost, and SVM, this study maximized remote-sensing data integration in determining soil bulk density as an important quality indicator of soil structure.

The difference in spectral band selection resulting from RF and XGboost is potentially attributable to the difference in RF and XGBoost algorithms. The individual trees in the RF model repetitively partitioned a random subset of the dataset into ever purer nodes (based upon the best random subset of predictors), and the results were then amalgamated into the ensemble. However, the boosting machines created an initial (usually relatively small) tree, shrank it, and then repeatedly partitioned the residuals of the previous tree; in essence, similar to incorporating partial regression into a decision tree. In this study, the XGBoost classifier performed slightly better than the RF and SVM classifier. Training data characteristics such as the number of training samples per class lead to performance differences of these algorithms [112]. For example, if RF and XGBoost were employed with unbalanced training data, the algorithms generally focus on the prediction accuracy of the prevailing classes, which might lead to lower accuracies in the less-represented classes. Low-bulk-density class size differs from high-bulk-density class size, which may lead to a low general prediction accuracy. For tackling this uncertainty, it is recommended that more field samples should be collected from the regions with a high bulk density to recalibrate or improve the models. Moreover, the tuning of hyperparameters has an impact on classification results and accuracies [113]. The slightly better performance of XGBoost than RF and SVM is due to the hyperparameters that were specifically used and tuned in the XGBoost algorithm. The hyperparameters, such as the learning rate, helped XGBoost algorithm repetitively leverage the patterns in residuals and improved model accuracy with the results from many decision trees generated in a sequential manner [114,115].

**Figure 7:**
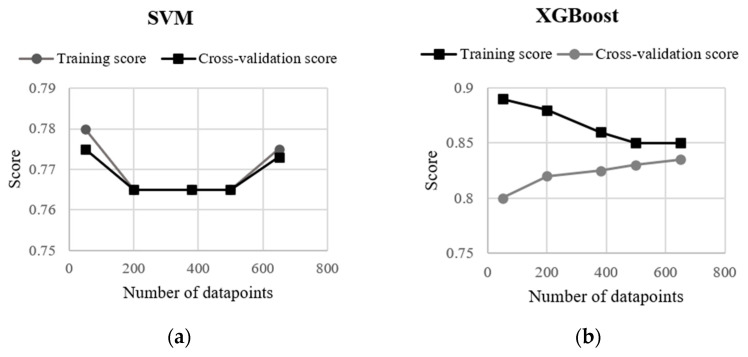
Learning curves on training and test datasets by (**a**) SVM and (**b**) XGBoost algorithms.

In Figure 7, a learning curve exhibits the validation and training scores of the XGBoost and SVM algorithms for varying numbers of training samples. For both models, the validation score and the training score converge to a value that was relatively high with the increasing size of the training set. The curves corresponding to the SVM model shows that adding more either training or testing data is not beneficial, although the training and validation scores were relatively high (0.78) at the beginning and the end of the curve, and the SVM model did not suffer from a variance error or a bias error. On the other hand, the XGBoost model shows an increase in the validation score as the number of data increases in the test dataset. Therefore, the curve suggests that a higher accuracy can be obtained from the confusion matrix by enlarging the test set. Although XGboost had the highest accuracy among the machine-learning algorithms used in this study, the difference in the accuracy resulted from RF, XGBoost and SVM were negligible (≤2%).

### 4.3. Uncertainties and Applications

Despite the importance of our results, it is also necessary to address the uncertainty in the sampling method of this research. The uncertainty may come from an artifact of absent data points related to soil bulk density of unvegetated areas. The high-bulk-density class defined in this study may not be detailed enough to distinguish the areas that are not ideal for vegetation growth due to extremely high salinity or bulk density (more than 1.400 g/cm^3^). In other words, this class includes a broad spectrum of soils having a bulk density greater than 0.752 g/cm^3^, and as such, it may not be exact in detecting areas that do not support any vegetation due to very high bulk density. However, the bulk density estimation performed in this study helps restoration scientists ensure that salt marsh soils are able to support specific vegetation at different sites across the Georgia coast. For example, low-bulk-density areas defined in this study tend to support salt marsh vegetation having very soft root structures such as *S. tabernaemontani,* while high-bulk-density areas are suitable for salt marsh vegetation such as *J. roemerianus* and *B. frutescens.* By using the models recommended by this study, it is feasible to understand which salt marsh species would be suitable for a restoration site from the perspective of soil structure indicator, i.e., soil bulk density. Figure 2 shows that *S. alterniflora* is able to grow in both low- and high-bulk-density salt marshes, and as such, soil classification in terms of bulk density may not be useful for determining sites suitable for *S*. *alterniflora* establishment and development along the Georgia coast.

In this study, bulk density data were collected from many well-distributed soil samples at a regional scale in order to detect and consider spatial variability in salt marsh soil bulk density along the Georgia coast. Regional-scale spatial variability in salt marsh soil bulk density across coastal Georgia can be described by diverse geomorphological units with distinct hydrological zones and vegetation communities. A better understanding of spatial variability in soil properties such as bulk density and organic matter content and their relationships with vegetation structure by using remote sensing will allow coastal resource scientists and managers to perform more reliable assessments and predictions of changes in salt marsh morphology.

## 5. Conclusions

The long-term monitoring and the continuous perseverance of salt marsh soil help the ecosystem maintain its ecological health and support its native flora and fauna. Understanding salt marsh soil’s physical properties, such as bulk density, guides ecologists and engineers to an effective restoration practice and a successful re-establishment of native vegetation. Machine-learning algorithms and Landsat-7 (ETM+) spectral bands were used in this study to model salt marsh soil bulk density. RF, SVM, and XGBoost were utilized to choose and rank the features with the highest efficiency to discriminate between the target classes through predictions obtained from an ensemble tree model. Among the machine-learning algorithms, XGBoost had the highest accuracy in classifying salt marsh soils into two main classes as low and high bulk densities. With the application of remote-sensing data and XGBoost algorithm, soil bulk density in salt marshes can be estimated, and the species of vegetation that are appropriate to survive in the estimated density level can be determined to expedite the restoration of salt marshes that are under anthropogenic and naturogenic disturbance regimes. Although this study illustrated the importance of the spectral resolution of a multispectral sensor in predicting soil bulk density, the effect of spatial resolution has not been investigated. It is assumed that greater spatial resolution will result in more accurate predictions if other sensor parameters are kept constant. Furthermore, we recommend hyperspectral data that provide very fine spectral bands in the VNIR and SWIR regions for future studies in order to determine the exact spectral bands useful for predicting salt marsh soil bulk density. In order to have a detailed classification or quantitative prediction of soil bulk density, a satellite sensor with high spectral and spatial resolution is preferred to create a soil bulk density map of a salt marsh site. A sensor with high spectral resolution introduces a large number of spectral features, which can be used as independent variables for quantitative and classification analysis in a machine-learning model. Therefore, the model will have a superior continuous prediction (high R square) or accurate classification (low MSE) of bulk density through the use of such a high dimensional dataset. A high dimensional dataset would provide more input covariable options to machine-learning algorithms through the selection and use of the features that have a strong influence on the variability in wetland soil bulk density. Moreover, conducting a time-series study to monitor salt marsh soil bulk density using remotely sensed data will help scientists detect changes in salt marsh conditions due to anthropogenic and naturogenic disturbances.

## Figures and Tables

**Table 1:** General statistical description of the dataset.

Data Source	Sampling Date	Number of Samples	Minimum	Maximum	Average	Standard Deviation
Our Survey	2018	24	0.17 g/cm^3^	1.66 g/cm^3^	0.78 g/cm^3^	0.51 g/cm^3^
CCRCN	2007–2013–2016–2018	622	0.18 g/cm^3^	1.56 g/cm^3^	0.62 g/cm^3^	0.43 g/cm^3^
GCE-LTER	2000–2009–2011	346	0.11 g/cm^3^	1.89 g/cm^3^	0.59 g/cm^3^	0.54 g/cm^3^

**Table 2:** SVM, RF, and XGboost models’ assessment results.

Models	Class	Recall	Precision	Mean Recall	Mean Precision	Accuracy
SVM	Low BD	0.96	0.87	0.78	0.84	0.86
	High BD	0.60	0.82			
RF	Low BD	0.88	0.96	0.85	0.79	0.87
	High BD	0.83	0.62			
XGBoost	Low BD	0.96	0.88	0.78	0.86	0.88
	High BD	0.61	0.84			

**Table 3:** Confusion matrix corresponding to the machine-learning algorithms.

SVM			
True			
Predicted		Low BD	High BD
	Low BD	178	25
	High BD	8	37
RF			
True			
Predicted		Low BD	High BD
	Low BD	179	25
	High BD	7	38
XGBoost			
True			
Predicted		Low BD	High BD
	Low BD	178	24
	High BD	8	39

## Data Availability

Not applicable.
